# Reference-free and cost-effective automated cell type annotation with GPT-4 in single-cell RNA-seq analysis

**DOI:** 10.21203/rs.3.rs-2824971/v1

**Published:** 2023-05-02

**Authors:** Wenpin Hou, Zhicheng Ji

**Affiliations:** 1 Department of Biostatistics, The Mailman School of Public Health, Columbia University, New York City, NY, USA; 2 Department of Biostatistics and Bioinformatics, Duke University School of Medicine, Durham, NC, USA.

## Abstract

Cell type annotation is an essential step in single-cell RNA-seq analysis. However, it is a time-consuming process that often requires expertise in collecting canonical marker genes and manually annotating cell types. Automated cell type annotation methods typically require the acquisition of high-quality reference datasets and the development of additional pipelines. We demonstrate that GPT-4, a highly potent large language model, can automatically and accurately annotate cell types by utilizing marker gene information generated from standard single-cell RNA-seq analysis pipelines. Evaluated across hundreds of tissue types and cell types, GPT-4 generates cell type annotations exhibiting strong concordance with manual annotations, and has the potential to considerably reduce the effort and expertise needed in cell type annotation.

In single-cell RNA-sequencing (scRNA-seq) analysis^1,2^, cell type annotation is a fundamental step to elucidate cell population heterogeneity and understand the diverse functions of different cell populations within complex tissues. Standard single-cell analysis software, such as Seurat^3^ and Scanpy^4^, routinely employ manual cell type annotation. These software tools assign single cells into clusters by cell clustering and conduct differential analysis to identify differentially expressed genes across cell clusters. Subsequently, a human expert compares canonical cell type markers with differential gene information to assign a cell type annotation to each cell cluster. This manual annotation approach requires prior knowledge of canonical cell type markers in the given tissues and is often laborious and time-consuming. Although several automated cell type annotation methods have been developed^5-13^, manual cell type annotation using marker gene information remains widely used in scRNA-seq analysis^14-28^.

Generative Pre-trained Transformers (GPT), including GPT-3, ChatGPT, and GPT-4, are large language models trained on massive amounts of data and capable of generating human-like text based on user-provided contexts. Recent studies have demonstrated the competitive performance of GPT models in answering biomedical questions^29-32^. Thus, we hypothesize that GPT-4, one of the most advanced GPT models, has the ability to accurately identify cell types using marker gene information. GPT-4 will potentially transform the manual cell type annotation process into a semi-automated procedure, with optional help from human experts to fine-tune GPT-4-generated annotations (Figure 1a). Compared to other automated cell type annotation methods that require building additional pipelines and collecting high-quality reference datasets, GPT-4 offers cost-efficiency and seamless integration into existing single-cell analysis pipelines, such as Seurat^3^ and Scanpy^4^. The vast amount of training data enables GPT-4 to be applied across a wide variety of tissues and cell types, overcoming the limitations of other automated cell type annotation methods restricted to specific reference datasets. Additionally, the chatbot-like nature of GPT-4 allows users to easily adjust annotation granularity and provide feedback for iterative answer improvement (Figure 1a-b)^31^.

To validate the hypothesis, we systematically assessed GPT-4’s cell type annotation performance across five datasets, hundreds of tissue types and cell types, and in both human and mouse (Figure 2a). Computationally identified differential genes in four scRNA-seq datasets (Azimuth by HuBMAP^22^, Human Cell Atlas (HCA)^17^, Human Cell Landscape (HCL)^19^, and Mouse Cell Atlas (MCA)^18^), and canonical marker genes identified through literature search in one dataset (literature)^17^, were used as inputs to GPT-4. Cell type annotation for HCL and MCA was performed and evaluated once by aggregating all tissues, similar to the original studies. In other studies, cell type annotation was performed and evaluated within each tissue. GPT-4 was queried using prompts similar to Figure 1b, and its cell type annotations were compared to those provided by the original studies. The comparison results were classified as “fully match” if GPT-4 and manual annotations refer to the same cell type, “partially match” if the two annotations refer to similar but distinct cell types (e.g., monocyte and macrophage), and “mismatch” if the two annotations refer to different cell types (e.g., T cell and macrophage). If the granularity of the manual annotation exceeded GPT-4 annotation, GPT-4 was asked to give more specific annotations (Figure 1b). Figure 2b shows an example of evaluating GPT-4 cell type annotations in a human prostate tissue literature search dataset. Supplementary Table 1 contains all cell type annotations generated manually or by GPT-4 across different tissue types and datasets, as well as agreement between manual and GPT-4 annotations.

The performance of cell type annotation can be affected by the number of top differential genes used as reference. So we first assessed whether the number of top differential genes would affect the performance of GPT-4 cell type annotation. To facilitate comparison, we assigned agreement scores of 1, 0.5, and 0 to cases of “fully match”, “partially match”, and “mismatch” respectively, and calculated the average scores across cell types within a tissue or dataset. The comparison was only performed in HCA, HCL, and MCA datasets, as full lists of differential genes were available. Figure 2c shows that GPT-4 has the best agreement with human annotation when using the top 10 differential genes, and using more differential genes may reduce agreement. A plausible explanation is that human experts may only rely on a small number of top differential genes if they already provide a clear cell type annotation. In subsequent analyses, we used GPT-4 cell type annotation with the top 10 differential genes for HCA, HCL, and MCA datasets.

In almost all studies and tissues, GPT-4 annotations fully or partially match manual annotations for at least 75% of cell types (Figure 2d), demonstrating GPT-4’s ability to generate cell type annotations comparable to those of human experts. The agreement is highest for marker genes identified through literature search, with GPT-4 annotations fully matching manual annotations for approximately 75% of cell types. The agreement decreases in marker genes identified by differential analysis, which may be attributable to a lower proportion of canonical marker genes being identified as top differential genes. We then grouped cell types into major cell categories according to the manual cell type annotations (Figure 2e, Supplementary Table 1). The agreement between GPT-4 and manual annotations is highest among cell categories that are more homogeneous (e.g., erythroid cells and adipocytes), and lowest among cell categories that are more heterogeneous (e.g., stromal cells).

The low agreement between GPT-4 and manual annotations in some cell types does not necessarily imply that GPT-4 annotation is incorrect. For instance, cell types classified as stromal cells include fibroblasts and osteoblasts, which express type I collagen genes, as well as chondrocytes, which express type II collagen genes. For cells manually annotated as stromal cells, GPT-4 assigns cell type annotations with higher granularity (e.g., fibroblasts, osteoblasts, and chondrocytes), resulting in partial matches and a lower agreement. For cell types manually annotated as stromal cells, the type I collagen genes appear in the differential gene lists in 80% of cases annotated as fibroblast or osteoblast by GPT-4 and in 0% of cases annotated as chondrocyte by GPT-4 (Figure 2f). This agrees with prior knowledge and the pattern observed in cell types manually annotated as chondrocyte, fibroblast, and osteoblast (Figure 2f), suggesting that GPT-4 provides more accurate cell type annotations than manual annotations for stromal cells.

We further tested the performance of GPT-4 when dealing with more complicated situations in real data analysis (Figure 1c). We first tested GPT-4’s ability to identify a cell cluster representing a mixture of cell types, which may occur when a cluster contains a large number of doublets or has low-resolution cell clustering. We generated simulated datasets by combining canonical markers from two distinct cell types in half of the instances and using canonical markers from a single cell type in the other half (Methods). GPT-4 discriminated between single and mixed cell types with an average accuracy of 94% (Figure 2g). We then tested GPT-4’s ability to identify new cell types with marker genes not documented by existing literature. We created simulation datasets using randomly selected genes as cell type markers in half of the cases and canonical markers from a single cell type in the other half (Methods). GPT-4 is able to differentiate known and unknown cell types with an average accuracy of 100% (Figure 2h). We also tested the reproducibility of GPT-4 annotations leveraging results in previous simulation studies (Methods). On average, GPT-4 generated identical annotations for the same cell type markers in 91.2% of cases (Figure 2i), showing a high level of reproducibility. In conclusion, GPT-4 exhibits robust performance across various scenarios encountered in real data analysis.

In conclusion, our findings demonstrate a high level of agreement between cell type annotations generated by GPT-4 and by human experts. Remarkably, GPT-4 exhibits higher accuracy in annotating specific cell types. GPT-4 can be employed as a dependable tool for automated cell type annotation of single-cell RNA-seq data, substantially reducing the time and effort required for manual annotation.

## Methods

### Dataset collection

For the HuBMAP Azimuth project, manually annotated cell types and their marker genes were downloaded from the Azimuth website (https://azimuth.hubmapconsortium.org/). Azimuth provides cell type annotations for each tissue at different granularity levels. We selected the level of granularity with the fewest number of cell types, provided that there were more than 10 cell types within that level.

For HCA^17^, HCL^19^, and MCA^18^, manually annotated cell types and corresponding differential gene lists were downloaded directly from the original studies. Lists of marker genes through literature search and the corresponding cell types were downloaded from the HCA study^17^, and only cell types with at least 5 marker genes were used.

### Gene set preparation and GPT-4 prompts

Before using GPT-4 to identify cell types, one needs to first prepare a list of top differential genes for each cell cluster. For example, one can use the following R code to extract gene lists of top 10 differential genes obtained from the standard Seurat pipeline. In the extracted results, each row is a list of differential genes for one cell cluster, separated by ’,’.


# d is the differential gene table generated by Seurat ordered by p-values cat(tapply(d$gene,list(d$cluster),function(i) paste0(i[1:10],collapse=‘,’)),sep=‘\n’)


The gene lists used in this study were prepared using customized code.

GPT-4 was accessed by visiting the ChatGPT website (https://chat.openai.com/). The “Mar 23” version of GPT-4 was used for this study. The following words were pasted on top of the differential gene lists and used as the initial prompt for GPT-4. The word “prostate” in the following prompt was replaced with the appropriate tissue names when annotating cell types for each tissue.


Identify cell types of human prostate cells using the following markers.
Identify one cell type for each row. Only provide the cell type name.


GPT-4 returned a list of cell type names for each query. The following prompt was used to increase the granularity of cell type annotations when needed.


Be more specific


To annotate cell clusters that could be a mixture of multiple cell types, the following words are added to the prompt.


Some could be a mixture of multiple cell types.


To annotate cell clusters that cannot be characterized by known cell type markers and are potentially new cell types, the following words are added to the prompt


Some could be unknown cell types.


Finally, the following prompt can be used to convert the list of cell type annotations generated by GPT-4 into R code that directly creates a vector of cell type names in R.


Use "‘,’" to concatenate all results into a single sentence.
Put "c(’" in front of the sentence and "’)" after the sentence


### Simulation studies and reproducibility

To generate simulation datasets of mixed cell types, we used the canonical cell type markers through literature search of human breast cells. In each simulation iteration, ten mixed cell types were generated. The marker genes for each mixed cell type were created by combining the marker gene lists of two randomly selected cell types. Additionally, we incorporated the original cell type markers of ten randomly chosen cell types as negative controls of single cell types. This entire simulation process was repeated five times. Subsequently, GPT-4 was queried using these simulated marker gene lists, and its performance in differentiating between mixed and single cell types was assessed.

To generate simulation datasets of unknown cell types, we compiled a list of all human genes using the Bioconductor org.Hs.eg.db package^33^. In each simulation iteration, ten simulated unknown cell types were generated. The marker genes for each unknown cell type were produced by combining ten randomly selected human genes. Additionally, we included the literature-based cell type markers of ten randomly chosen human breast cell types as negative controls of known cell types, similar to the previous simulation study. This entire simulation process was repeated five times. Subsequently, GPT-4 was queried using these simulated marker gene lists, and its performance in distinguishing between known and unknown cell types was assessed.

We assessed the reproducibility of GPT-4 responses by leveraging the repeated querying of GPT-4 with identical marker gene lists of the same negative control cell types in both simulation studies. For each cell type, reproducibility is defined as the proportion of instances in which GPT-4 generates the most prevalent cell type annotation. For instance, in the case of vascular endothelial cells, GPT-4 produces "endothelial cells" 8 times and "blood vascular endothelial cells" once. Consequently, the most prevalent cell type annotation is "endothelial cells," and the reproducibility is calculated as $\frac{8}{9}=0.89$.

## Figures and Tables

**Figure 1.**
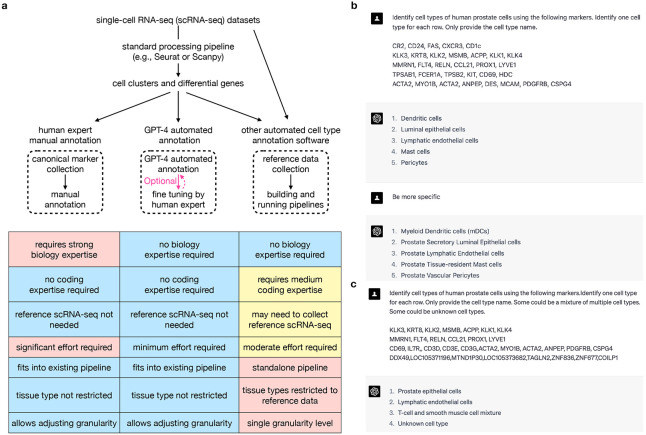
**a,** Diagram comparing cell type annotations by human experts, GPT-4, and other automated methods. **b,** An example showing GPT-4 prompts and answers for annotating human prostate cells with increasing granularity. **c,** An example showing GPT-4 prompts and answers for annotating single cell types (first two cell types), mixed cell types (third cell type), and new cell types (fourth cell type).

**Figure 2.**
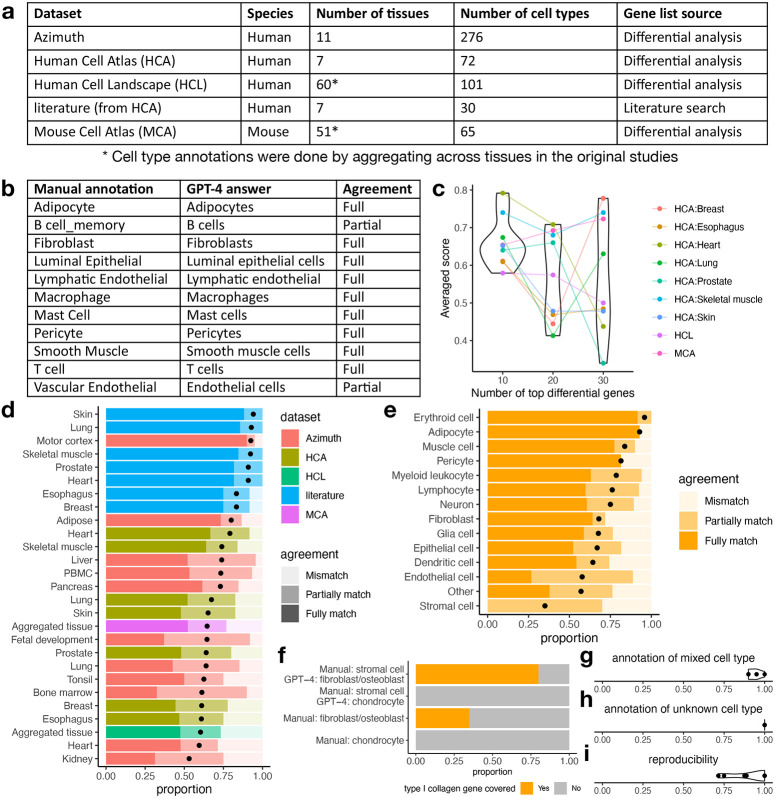
Evaluation of cell type annotation by GPT-4. **a,** Datasets included in this study **b,** Agreement between original and GPT-4 annotations in identifying cell types of human prostate cells. **c,** Averaged agreement score (y-axis) and the number of top differential genes (x-axis) in HCA, HCL, and MCA datasets. **d,** Proportion of cell types with different levels of agreement in each study and tissue. Averaged agreement scores are shown as black dots. **e,** Proportion of cell types with different levels of agreement in each cell category. Averaged agreement scores are shown as black dots. **f,** Proportion of cell types that include type I collagen gene in the differential gene lists. The cell types are either classified as stromal cells by manual annotations and fibroblast, osteroblast, or chondrocyte by GPT-4 annotations, or classified as fibroblast, osteroblast, or chondrocyte by manual annotations. **g,** Proportion of cases where GPT-4 correctly identifies mixed and single cell types. Each dot represents one round of simulation. **h,** Proportion of cases where GPT-4 correctly identifies known and unknown cell types. Each dot represents one round of simulation. **i,** Reproducibility of GPT-4 annotations. Each dot represents one cell type.
